# Detection of OqxAB Efflux Pumps, a Multidrug-Resistant Agent in Bacterial Infection in Patients Referring to Teaching Hospitals in Ahvaz, Southwest of Iran

**DOI:** 10.1155/2021/2145176

**Published:** 2021-11-22

**Authors:** Mojtaba Moosavian, Mahtab Khoshkholgh Sima, Nazanin Ahmad Khosravi, Effat Abbasi Montazeri

**Affiliations:** ^1^Infectious and Tropical Diseases Research Center, Health Research Institute, Ahvaz Jundishapur University of Medical Sciences, Ahvaz, Iran; ^2^Department of Microbiology, School of Medicine, Ahvaz Jundishapur University of Medical Sciences, Ahvaz, Iran

## Abstract

Antibiotic resistance mechanisms in Enterobacteriaceae are causative agents of global health problems. Bacterial infections due to multidrug resistance (MDR) may be mediated by the overexpression of efflux pumps. In this study, we investigated the prevalence of o*qxA* and o*qxB* genes as two encoding agents of efflux pumps and the determination of antibiotic resistance rate in clinical isolates of Enterobacteriaceae. In this study, 100 Enterobacteriaceae isolates collected from different clinical specimens of infectious patients, such as wounds, urine, blood, discharge, and abscesses except stool, were examined. Identification of the isolates was performed using standard biochemical tests such as TSI, citrate, urea, lysine, SIM, MR-VP, and gas production. The antimicrobial susceptibility test was carried out by the Kirby–Bauer disk diffusion method according to CLSI guidelines, and finally, the *oqxA* and *oqxB* genes were detected by the PCR method. Among 100 Enterobacteriaceae isolates, *Escherichia coli* and *Enterobacter gergoviae* were the most common isolates with 71% and 20%, respectively. Also, the lowest isolates belonged to *Enterobacter cloacae* (3%) and *Klebsiella pneumoniae* (1%). Out of 100 Enterobacteriaceae isolates, 37 isolates (37%) were positive for at least one of o*qxA* or *oqxB* genes, while both of these genes were detected among 12% of them. *oqxAB* genes were detected in 8 cases of 20 (40%) *Enterobacter gergoviae* and 4 cases of 71 (5.7%) *E. coli* isolates. The antimicrobial susceptibility test showed that all isolates (100%) were susceptible to imipenem, while the maximum resistance to piperacillin, ceftriaxone, and cefotaxime were 69%, 55%, and 55%, respectively. Also, the results of this study showed that antibiotic resistance in Enterobacteriaceae isolates caused by oqxAB genes is increasing among patients in Iran. Therefore, identification of resistant isolates and antibiotic monitoring programs are essential to prevent the spread of MDR isolates.

## 1. Introduction

Bacterial multidrug resistance (MDR) is an increasing problem in healthcare in both the hospital and community settings [1]. Gram-negative bacilli (GNB) resistance is a challenge for intensive care unit (ICU) physicians. GNB causes 45–70% of ventilator-associated pneumonia (VAP), 20–30% of catheter-related sepsis, and other ICU-acquired sepsis [2]. Mechanisms dependent on changes in membrane permeation processes has been reported as cause contributors of MDR [3]. At present time, it has been known that the plasmid-encoded OqxA and OqxB pumps confer resistance to multiple antimicrobial agents, such as quinolones (example, nalidixic acid) and fluoroquinolones (example, ciprofloxacin, norfloxacin, and flumequine) as well as biocides such as triclosan and chlorhexidine [4, 5].

The extended-spectrum cephalosporins and fluoroquinolones have been known as the choice drugs for the treatment of acute gastroenteritis caused by enteric pathogens. It has been now cleared that the resistance of these organisms to antimicrobials such as fluoroquinolone and quinolone could be due to mutation in the target region of quinolone resistance determining in DNA gyrases and type IV topoisomerases which ultimately prevent drugs from binding to this target [6].

Also, some of the reports show the multidrug efflux mechanisms that are widely conserved in bacteria are almost encoded by chromosomes. On the other hand, specific drug efflux mechanisms are usually encoded by plasmids and/or other mobile genetic elements (transposons and integrons) that carry resistance genes [7]. Pumps may transport a wide range of unlike compounds (including several classes of antibiotics). Such pumps can be associated with MDR [8].

The OqxAB efflux pump, a plasmid-mediated quinolone resistance (PMQR) element, has become widespread among members of Enterobacteriaceae over the past decade [9]. OqxAB pumps are encoded by two *oqxA* and *oqxB* genes, which are localized in one operon. The OqxAB efflux pumps cause resistance to fluoroquinolones (FQ) and other agents [10].

However, antibiotic resistance among some clinical isolates of Enterobacteriaceae such as *E. coli*, *Klebsiella pneumoniae*, and *Salmonella* are important not only in humans but also among animals, such as horses, swine, pork, and pigs, because some resistant agents such as the OqxAB pump encoded by plasmids (as pOLA 52), which harbored in these animals [6].

In this study, we investigated the prevalence of o*qxA* and o*qxB* genes and the determination of antibiotic resistance rate in Enterobacteriaceae isolates collected from clinical specimens of infectious patients.

## 2. Materials and Methods

### 2.1. Ethics Consideration

Ethics approval and consent to participate are not applicable in this study.

### 2.2. Bacterial Isolates from Specimens

In this study, 100 Enterobacteriaceae isolates from clinical specimens (wound, urine, blood, discharge, and abscesses except stool) which were collected by hospital laboratories from infectious patients were examined. These patients were referred to Golestan and Imam Khomeini Teaching Hospitals related to Ahvaz Jundishapur University of Medical Sciences, Ahvaz, Iran. After reculture on blood agar and MacConkey agar media, the isolates were identified by standard biochemical tests such as triple sugar iron agar (TSI), citrate, urea, lysine, SIM, MR-VP, and gas production (all culture media were provided by Merck, Germany) [11].

### 2.3. Antimicrobial Susceptibility Test

All isolates were subjected to antimicrobial sensitivity testing by the Kirby–Bauer disk diffusion method on Mueller–Hinton agar (MHA) (Merck, Germany) according to the Clinical and Laboratory Standard Institute (CLSI-2015) guidelines [12]. The tested antimicrobial agents were as follows: imipenem (10 *μ*g), ceftriaxone (30 *μ*g), ceftazidime (30 *μ*g), piperacillin (100 *μ*g), ciprofloxacin (5 *μ*g), gentamicin (10 *μ*g), amikacin (30 *μ*g), cefotaxime (30 *μ*g), norfloxacin (10 *μ*g), nalidixic acid (30 *μ*g), and levofloxacin (5 *μ*g). The antibiotic disks provided from MAST Group Ltd., Merseyside, U.K.

### 2.4. DNA Extraction and PCR of the *oqxA* and *oqxB* Genes

DNA template was prepared by the boiling method [13]. Briefly, 3-4 bacterial colonies were suspended in 500 *μ*l TE buffer. The samples were incubated at 95°C for 15 minutes. Then, they were centrifuged for 10 minutes at 4°C and 12000 rpm, and the supernatants were stored in Eppendorf microtubes at −20°C, which were used as DNA templates. The concentration of the extracted DNA was measured by a photometer (Eppendorf, Germany) in 260/280 nm UV long waves. PCR master mixture was prepared in each 25 *μ*l reaction containing 2.5 *μ*l in 10X PCR buffer, 0.5 *μ*l of dNTP mix (10 mM), 0.75 *μ*l MgCl_2_ (50 mM), 1 *μ*l of each primer (10 pmol) TAG, A/S Denmark (Table 1), 0.25 *μ*l of Taq DNA polymerase (5 U/*μ*l), 1 *μ*l of DNA template, and 18 *μ*l of distilled water.

DNA amplification of the o*qxA* gene was performed in a thermocycler (Eppendorf, Germany) under initial denaturation at 94°C for 5 min, followed by 34 cycles of denaturation at 94°C for 45 s, annealing at 51°C for 45 s, extension at 68°C for 1 min, and the final step of extension at 72°C for 10 min. These conditions for the *oqxB* gene were 94°C for 45 s (denaturation) followed by 32 cycles, 64°C for 45 s, and 72°C for 60 s [14].

### 2.5. Electrophoresis

The PCR product was electrophoresed on 1.5% agarose gel (Cinna GenCo, Iran) in 1X buffer Tris/borate/EDTA buffer (Cinna GenCo, Iran) at 120 V for 60 minutes. The DNA was stained with ethidium bromide (Cinna GenCo, Iran), and photography of DNA amplified was performed in gel documentation (Viber Company, France). In this study, *Klebsiella pneumoniae* ATCC 700603 [4] and *E. coli* ATCC 25922 [14] were used as the positive control and negative control, respectively.

## 3. Statistical Analysis

To analyze the data, a chi-squared test was run in SPSS version 16 (SPSS Inc., Chicago, IL, USA). *P* value <0.05 was considered statistically significant.

## 4. Results

Out of 100 Enterobacteriaceae isolates, the most isolates belonged to *Escherichia coli* (71%), *E. gergoviae* (20%), *E. cloacae* (3%), *E. aerogenes* (5%), and *K. pneumoniae* (1%). The highest resistance was observed for piperacillin (69%), ceftriaxone (55%), cefotaxime (55%), ceftizoxime (43%), ceftazidime (42%), nalidixic acid (39%), ciprofloxacin (36%), norfloxacin and gentamicin (26%), and levofloxacin (23%). The lowest resistance was observed for imipenem (0%) and amikacin (5%). The resistance rates to antimicrobial agents are given in Table 2.

**Figure 1:**
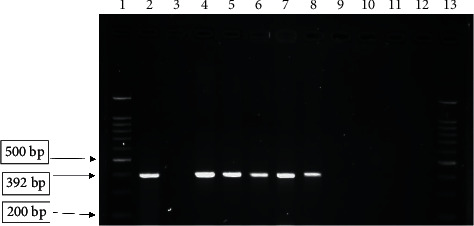
Electrophoresis of o*qxA* gene PCR product. Lanes 1 and 13, 100 bp DNA ladder; lane 2, positive control (*Klebsiella pneumoniae* ATCC 700603); lane 3, negative control (*E coli* ATCC 25922); lanes 4–8, positive samples for *oqxA*; lanes 9–12, negative samples.

**Figure 2:**
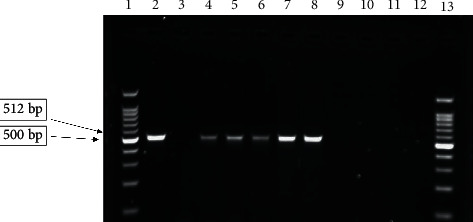
Electrophoresis of o*qxB* gene PCR product. Lanes 1 and 13, 100 bp DNA ladder; lane 2, positive control (*Klebsiella pneumoniae* ATCC 700603); lane 3, negative control (*E. coli* ATCC 25922); lanes 4–8, positive samples for *oqxB*; lanes 9–12, negative samples.

PCR results showed that out of 100 Enterobacteriaceae isolates, 37 isolates were positive for at least one of both genes. Indeed, the prevalence of *oqxA* and *oqxB* genes among Enterobacteriaceae isolates was 22% and 15%, respectively (Figures 1 and 2), while both o*qxA* and o*qxB* genes were found in 12% of them (*P* > 0.05). These results showed that all of positive results of efflux pump were related to the ciprofloxacin- and norfloxacine-resistant strains, and also, all of the susceptible isolates to the ciprofloxacin and norfloxacine were negative for efflux pump genes. *oqxAB* genes were detected in 40% (8 of 20) of *E. gergoviae* and 5.7% (4 of 71) of *E. coli* isolates.

## 5. Discussion

Bacterial multidrug resistance may be mediated by the overexpression of efflux pumps [15]. In MDR bacteria, overexpression of efflux pumps leads to reduced drug sensitivity by decreasing the intracellular concentration of antibiotics [3]. Dissemination of *oqxAB* genes may pose a great risk to food safety and public health [16].

In this study, we detected *oqxAB* genes in clinical isolates of Enterobacteriaceae. Since bacterial resistance to antibiotics is related to different agents, in this study, detection of efflux pump genes was performed by PCR on all isolates to determine whether their resistance to antibiotics, including ciprofloxacin and norfloxacine, depended on the presence of these genes or other agents. Analysis of PCR results showed that although 37% of the isolates were positive for at least one *oqxA* or *oqxB* gene, the prevalence of both *oqxA* and *oqxB* was not significantly different between Enterobacteriaceae isolates (*P* > 0.05). Also, isolates sensitive to antibiotics, including ciprofloxacin and norfloxacine, lacked the oqxAB pump, while resistance isolates without efflux pump may be affected by other factors, such as reduced antibiotic permeability, decreased accumulation of intracellular antibiotics, and inactivation of the drugs. The incidence of *oqxAB* genes was 40% and 5.7% for *E*. *gergoviae* and *E. coli*, respectively. Yuan et al. (2012) showed that *oqxB* and *oqxB* genes were present in 6.6% of *E. coli* strains [17]. Kao et al. (2016) reported *oqxAB* genes in 6.05% of *E. coli* isolates [18].

Kim et al. (2009) investigated the prevalence of plasmid-encoded multidrug efflux pump in clinical isolates of Enterobacteriaceae. In their survey, 0.4% of *E. coli* isolates and 4.6% of *E. cloacae* isolates were positive for both *oqxB* and *oqxB* genes [14]. Because these studies were performed at different times, therefore, it could be the reason for different results. Zhao et al. (2010) reported the *oqxA* gene in 30.3% of *E. coli* isolates which were collected from farmworkers [19]. In our study, all *oqxAB*-positive Enterobacteriaceae strains were resistant to piperacillin, ceftriaxone, cefotaxime, ceftizoxime, ciprofloxacin, and ceftazidime, while all of these isolates (100%) were susceptible to imipenem.

As some reports have confirmed that OqxA and OqxB efflux pumps confer resistance to multiple antimicrobial agents (4, 5), therefore, in our study also, the presence of OqxAB efflux pumps in clinical isolates of Enterobacteriaceae containing one or both *oqxA* and *oqxB* genes has conferred resistance to multiple antimicrobial agents.

In this study, the highest resistance was observed for piperacillin, ceftriaxone, and cefotaxime (≥55%), while Tang et al. (2016) showed that Enterobacteriaceae clinical isolates (carbapenem-resistant) were more resistant to some antibiotics, for example, their resistance (nonsusceptible) to cefuroxime, ceftazidime, and ampicillin was 100% and to piperacillin was 88.5% (20). In contrast to the study of Tang et al. (2016), which showed that the lowest resistance of these isolates to imipenem and amikacin was 32 and 20.5, respectively [20], in our study, the lowest resistance rate for imipenem and amikacin was 0% and 5%, respectively. However, Ye et al. (2018) showed that 83.3% of Enterobacteriaceae clinical isolates (carbapenem-resistant) were simultaneously resistant to imipenem, meropenem, and ertapenem [21].

Different resistance rates in various studies could be due to carrying two or more ESBLs, *oqxAB*, and carbapenemase genes or a combination of these genes in Enterobacteriaceae isolates.

In our study, the *oqxA* and *oqxB* genes were not detected in *K. pneumoniae*, *E. cloacae*, and *E. aerogenes* isolates, whereas Park et al. (2012) showed *oqxAB* genes in 14.4% of *K. pneumoniae* isolates, which were resistant to nalidixic acid, olaquindox, levofloxacin, and ciprofloxacin [5]. The results of our study also indicated that all *oqxAB*-positive *E. coli* strains and 75% of *oqxAB*-positive *E. gergoviae* strains were resistant to nalidixic acid. These results suggest that the presence of *oqxAB* genes may be related to the resistance of these isolates to quinolone.

## 6. Conclusion

The results of this study showed that Enterobacteriaceae isolates with efflux pumps such as o*qxAB* are increasing in Iran. Therefore, identification of resistant isolates and antibiotic monitoring programs are essential to prevent the spread of MDR isolates.

## Figures and Tables

**Table 1:** Primers used in this study.

Genes	Primer sequences	Product size (bp)	References
*oqxA*	oqxAF-5′-CTCGGCGCGATGATGCT-3′	392	14
	oqxAR-5′-CCACTCTTCACGGGAGACGA-3′		
*oqxB*	oqxBs-5′-TTCTCCCCCGGCGGGAAGTAC-3′	512	
	oqxBa2-5′-CTCGGCCATTTTGGCGCGTA-3′		

**Table 2:** The results of the antibiogram test for Enterobacteriaceae isolates.

		Resistant (%)											
Bacteria	Number	IMP	CRO	CZX	GM	CP	PIP	AN	CAZ	CTX	NA	NOR	LEV
*E. coli*	71	0	39	26	18	27	52	1	28	38	33	21	20
*Enterobacter gergoviae*	20	0	9	10	6	9	12	4	9	14	6	5	3
*Enterobacter cloacae*	3	0	3	2	1	0	2	0	3	1	0	0	0
*Enterobacter aerogenes*	5	0	4	5	1	0	3	0	2	2	0	0	0
*Klebsiella pneumoniae*	1	0	0	0	0	0	0	0	0	0	0	0	0
Total	100	0	55	43	26	36	69	5	42	55	39	26	23

IMP, imipenem; CRO, ceftriaxone; CZX, ceftizoxime; GM, gentamicin; CP, ciprofloxacin; PIP, piperacillin; AN, amikacin; CAZ, ceftazidime; CTX, cefotaxime; NA, nalidixic acid; NOR, norfloxacin; LEV, levofloxacin.

## Data Availability

The data generated or analyzed during this study are included within this article.

## Additional Points

Our study has the following limitations. For example, the different patterns of antibiotic resistance in each efflux pump and strain typing of isolates were not determined, while the presentation of them could help with more information for readers.
